# Effects of Understory Interplanting of Broad-Leaved Trees on a *Pinus massoniana* Plantation’s Biomass and Soil Stoichiometric Characteristics in Southern China

**DOI:** 10.3390/plants15213456

**Published:** 2026-11-05

**Authors:** Hui Hu, Nana Li, Yanbing Yan, Yongsheng Cui, Xiaolong Hou, Pengfei Wu

**Affiliations:** 1 College of Forestry, Fujian Agriculture and Forestry University, Fuzhou 350002, China; huhui5258548@163.com (H.H.); fafulinana@126.com (N.L.); cuiys2023@fafu.edu.cn (Y.C.); xl.hou@fafu.edu.cn (X.H.); 2 Colleges and University Engineering Research Center of Plantation Sustainable Management, Fuzhou 350002, China; 3 Key Laboratory of State Forestry and Grassland Administration on Soil and Water Conservation of Red Soil Region in Southern China, Fuzhou 350002, China; 4 Baisha State-Owned Forest Farm, Shanghang County, Longyan 364200, China; 19856169319@163.com

**Keywords:** uneven-aged multi-layered forest, *Pinus massoniana*, biomass, understory vegetation, stoichiometric characteristics

## Abstract

In the context of global warming and declining forest ecosystem services, this study explores underplanting effects on *Pinus massoniana* stand biomass allocation and soil C–N–P stoichiometry to support sustainable plantation management in southern China. Two underplanting treatments were established: *P. massoniana* underplanted with *Schima superba* (denoted by PS), and *P. massoniana* underplanted with *S. superba* + *Michelia macclurei* + *Castanopsis hystrix* (denoted by PSMC). A pure *P. massoniana* stand was set as the control (CK). The tree height, DBH and soil C–N–P stoichiometric characteristics were measured, and we compared biomass distribution among the tree layer, understory vegetation layer and litter layer. The results showed that compared with CK and PS, the PSMC significantly promoted the height and DBH growth of *P. massoniana* and substantially increased the biomass of all tree (leaves, branches, stems, and roots), sustaining higher stand productivity. In contrast, underplanted *S. superba* inhibited the growth of *P. massoniana*. Both underplanting treatments significantly reduced understory vegetation biomass, with herbs dominating the understory layer in both treatments. Litter biomass was the lowest under the PS treatment and was significantly lower than others. Compared with CK, both the PS and PSMC treatments significantly increased soil C, N, and P contents as well as soil C:P and N:P ratios in the 0–20 cm and 20–40 cm soil layers. The DBH of *P. massoniana* was positively correlated with soil C, N, and P concentrations in the 0–20 cm and 20–40 cm soil layers, and it exhibited an extremely significant positive correlation with soil N concentration and soil N:P ratio in the 40–60 cm soil layer. Tree-layer biomass was significantly increased with soil P concentration and the N:P ratio in the 0–20 cm soil layer, and it showed an extremely significant positive correlation with soil C and N concentrations in the 0–20 cm and 20–40 cm soil layers, as well as with soil N concentration and the N:P ratio in the 40–60 cm soil layer. In addition, litter-layer biomass was significantly and positively correlated with soil P concentration in the 20–40 cm soil layer. This association occurs because mixed underplanted broadleaves capture phosphorus released by litter decomposition via their well-developed roots and redistribute P to subsoil, and subsoil P availability controls litter production by regulating tree nutrient status. In conclusion, compared with CK, PSMC significantly improved *P. massoniana* individual growth and tree-layer biomass, while decreasing understory vegetation biomass and raising the 40–60 cm soil N:P ratio. Deep-soil N:P ratio was positively correlated with tree-layer biomass, implying that PSMC supports high stand productivity.

## 1. Introduction

Forests serve as a critical carbon pool in terrestrial ecosystems. Plantations are established via artificial planting or direct seeding and have increasingly become an important component of forest ecosystems worldwide. Over the past two decades, large-scale afforestation in China has contributed approximately 25% to the global expansion of green-covered area [1,2]. The total area of plantations in China has reached 66 million ha, and nearly 50% of these are monoculture plantations. These monoculture plantations have, to a certain extent, promoted social production and economic development [3]. However, unreasonable and extensive monoculture plantation management may trigger multiple adverse ecological consequences, including biodiversity loss, soil degradation, water scarcity, unstable carbon sequestration capacity, and aggravated climate change [4,5,6,7]. Under the dual pressures of global climate warming and the degradation of forest ecosystem services, enhancing forest biodiversity and improving soil nutrient conditions of plantations have become a key task for ecological restoration [8]. Introducing native broad-leaved tree species to construct mixed stands is widely regarded as an effective strategy to solve the above problems.

Numerous studies have demonstrated that introducing native broad leaved tree species to construct uneven aged multi layered stands can optimize stand structure, substantially improve stand growth, and effectively boost ecosystem services [9]. Compared with pure stands, mixed species stands generally achieve higher productivity, which is largely attributed to species complementarity effects. However, the impacts of species selection and collocation for underplanting on the productivity of plantation stands are inconsistent [10]. Zhang et al. [11] compared different conifer–broadleaf mixed forests transformed from pure *Cunninghamia lanceolata* stands and found that the biomass of each plant organ and total stand biomass varied significantly with the configuration patterns of broad-leaved tree species. Such differences in biomass are closely associated with soil nutrient status regulated by mixed tree species. Soil carbon (C), nitrogen (N), and phosphorus (P) stoichiometric characteristics reflect soil nutrient supply status of forest stands and act as key limiting factors for stand productivity [12]. Different configuration patterns of conifer–broadleaf mixed tree species can indirectly affect plant biomass by regulating soil nutrient contents. Studies have shown that different configuration patterns of conifer-broadleaf mixed tree species can indirectly regulate soil nutrient availability by altering nutrient uptake and utilization of plant communities. Furthermore, compared with monoculture and two-species mixed plantations, multi-species mixed forests exhibit prominent advantages in soil organic carbon accumulation, which is predominantly modulated by soil available nitrogen and potassium contents, as well as changes in soil enzyme activities [13]. Thus, taking soil stoichiometric characteristics as an entry point to elucidate how different understory interplanting patterns influence stand level productivity and biomass allocation can help identify optimal interplanting patterns to improve stand productivity and stability.

*Pinus massoniana* is widely planted across Southern China and has high economic value; its fast-growing trait renders it instrumental in early-stage ecological restoration and forestry economic development [14]. Nevertheless, long-term monoculture management has resulted in prominent problems including frequent pest and disease outbreaks, reduced stand productivity, and ecological imbalance, which limits the ecological functions of *P. massoniana* plantations [15]. Liu et al. [16] verified that interplanting broad-leaved tree species within *P. massoniana* plantations can not only enrich community composition but also promote stand growth and soil nutrient amelioration. However, understory interplanting alters canopy microclimates and creates persistent low light conditions within stands, which in turn suppresses the early growth of newly introduced broad-leaved species such as *Phoebe bournei* [17]. Accordingly, rational selection and interplanting of native broad-leaved tree species are critical for sustaining the long-term productivity of *P. massoniana* plantations. To this end, three major native broad-leaved tree species were selected, including *Schima superba*, *Michelia macclurei*, and *Castanopsis hystrix*. Two interplanting treatments were set: *P. massoniana* interplanted with *S. superba* (PS), and *P. massoniana* interplanted with *S. superba* + *M. macclurei* + *C. hystrix* (PSMC), with a pure *P. massoniana* stand serving as the control (CK). We quantified the biomass of the tree layer, understory vegetation layer, and litter layer, as well as soil C, N, and P stoichiometric characteristics across all three treatments. The objectives were as follows: (1) to explore the effects of different understory interplanting patterns on soil C, N, and P concentrations and their stoichiometric ratios in *P. massoniana* plantations; (2) to reveal stand biomass allocation patterns of *P. massoniana* plantations under different understory interplanting patterns.

## 2. Results

### 2.1. Effects of Understory Interplanting on P. massoniana Growth

**Figure 1:**
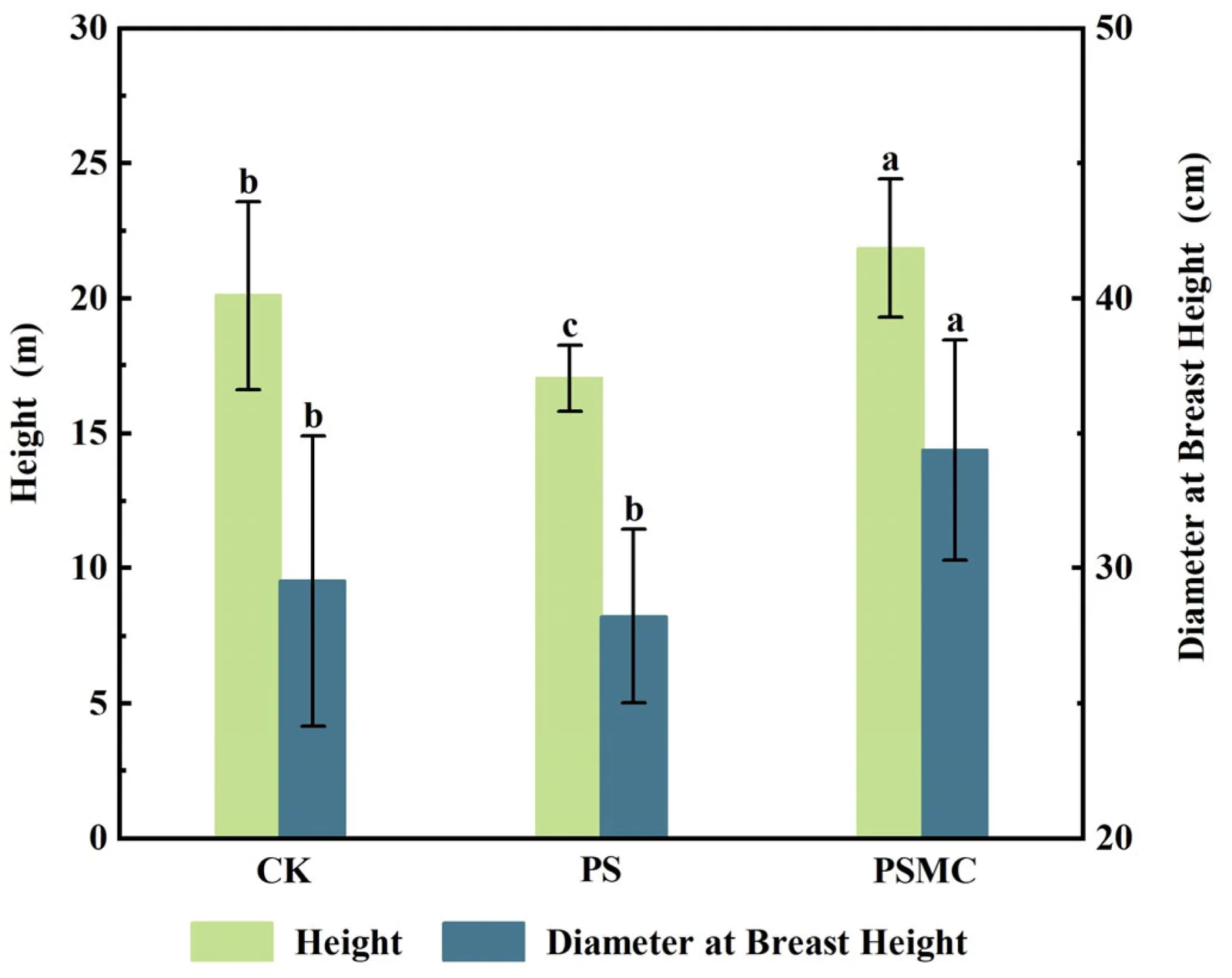
Variations in tree height and DBH of *P. massoniana* in different treatments. Data are presented as mean ± SE. Different lowercase letters indicate significant differences between different forest stands (*p* < 0.05).

Trees of *P. massoniana* under the PSMC showed the maximum height and diameter at breast height (DBH), which were significantly greater than those in the PS and CK (*p* < 0.05). By contrast, the PS stand yielded the minimum values for both tree height and DBH. Specifically, the DBH of *P. massoniana* in the PSMC stand was 16.50–21.86% higher than others. Meanwhile, tree height under the PSMC treatment was 28.24% significantly greater than that in PS treatment and 8.70% greater than that in CK. These results suggested that understory interplanting of *S. superba*, *C. hystrix*, and *M. macclurei* within *P. massoniana* plantations exerted positive effects on height–DBH growth of *P. massoniana* (Figure 1).

### 2.2. Effects of Understory Interplanting Patterns on the Biomass of Different Components in P. massoniana Plantations

**Figure 2:**
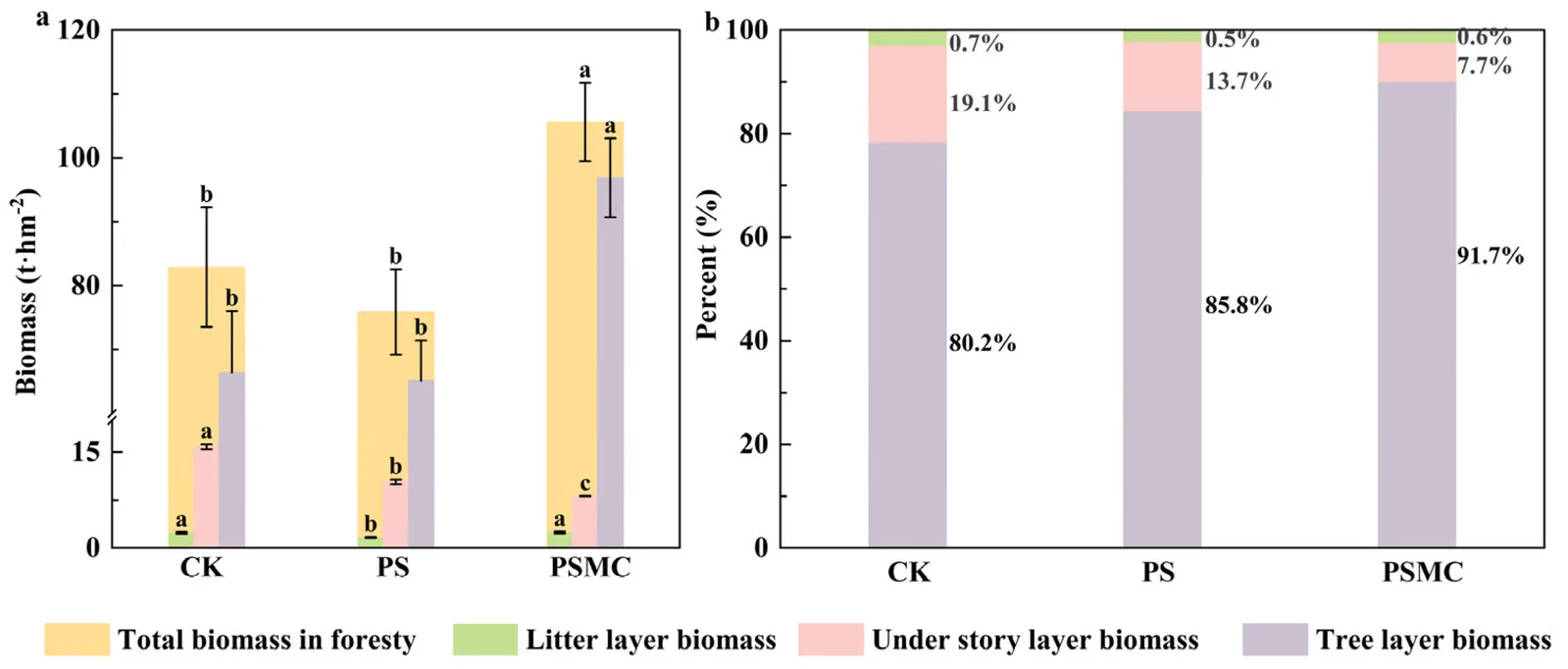
Biomass and proportion of each component in different treatments. (**a**) Stand biomass of each layer. (**b**) Proportion of biomass in each stand layer. Data are presented as mean ± SE. Different lowercase letters indicate significant differences between different forest stands (*p* < 0.05).

The total biomass and tree-layer biomass in PSMC stand were significantly greater than those in PS and CK stands, with tree-layer biomass increased by 48.78% and 45.82% relative to the PS and CK stands, respectively. Understory vegetation biomass in the PS and PSMC stands declined by 34.57% and 48.71%, respectively, compared with CK. The PSMC stand had the highest litter-layer biomass (in 2.44 t·hm^−2^) (Figure 2a). Across the three experimental stands, the biomass-allocation pattern generally exhibited the following proportions; the tree-layer accounted for 80.16–91.72%, the understory vegetation layer for 7.7–19.14%, and the litter-layer for 0.53–0.70%. Compared to CK, the proportion of tree-layer biomass in the PSMC stand increased by 14.42%, while the proportion of understory vegetation biomass declined by 59.77% (Figure 2b). These results suggest that the multi-species interplanting regime improved total stand biomass by optimizing the biomass-allocation pattern.

As shown in Table 1, the biomass of each tree-layer organ ranked in stem > root > branch > leaf. The biomass of all tree-layer organs in the PSMC stand was significantly greater than that in the CK and PS stands. The proportions of above-ground biomass (leaf + branch + stem) in CK, PS, and PSMC stands were 82.27%, 82.15%, and 81.67%, respectively, exhibiting a declining trend. Meanwhile, the root biomass of the PSMC stand reached 17.76 t·hm^−2^, which was 50.76% and 52.84% greater than that in the CK and PS stands, respectively. The corresponding proportions of root biomass to total tree biomass were 17.73%, 17.85%, and 18.33% for CK, PS, and PSMC stands, respectively, showing only a slight increase. These findings suggested that understory interplanting of broad-leaved species promoted root growth and the accumulation of absolute root biomass, while the relative biomass allocation to roots remained largely unchanged.

**Figure 3:**
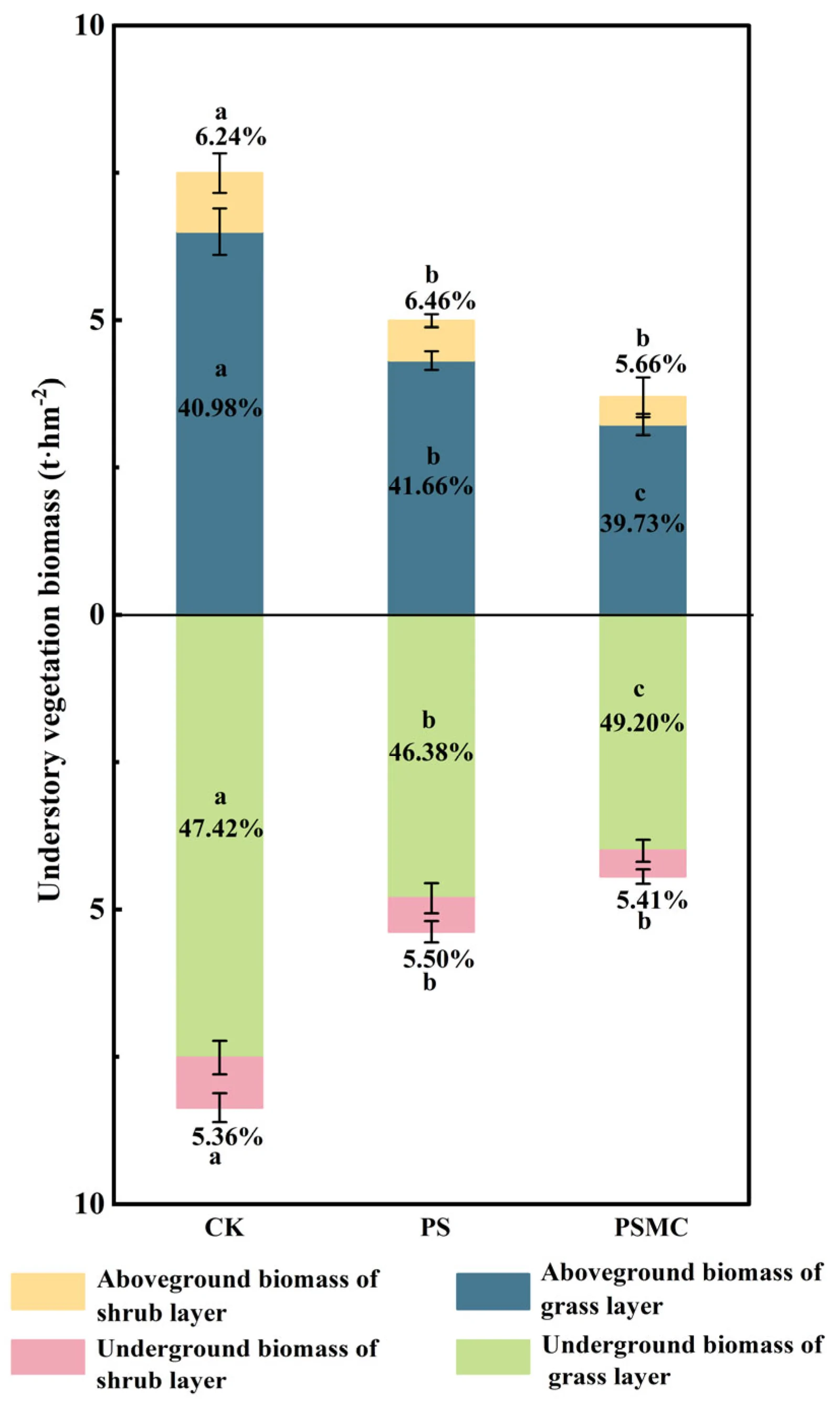
Biomass and distribution of understory vegetation layer in different treatments. Data are presented as mean ± SE. Different lowercase letters indicate significant differences between different forest stands (*p* < 0.05).

As shown in Figure 3, the total understory vegetation biomass differed significantly among the three stand types (*p* < 0.05). The highest value was detected in CK (15.85 t·hm^−2^), followed by the PS stand (accounting for 65.38% of that in CK), while the lowest value occurred in the PSMC stand (8.13 t·hm^−2^).

Furthermore, herb-layer biomass was greater than shrub-layer biomass, and the above-ground biomass of understory vegetation was slightly less than its below-ground biomass. Shrub-layer biomass under both the PS and PSMC treatments was significantly lower than that under CK. These results suggested that understory interplanting of broad-leaved species suppressed understory vegetation growth, and that the herb layer held a competitive advantage over the shrub layer under the interplanting regimes.

### 2.3. Effects of Understory Interplanting Patterns on Soil Nutrient Contents and Their Stoichiometric Characteristics

**Figure 4:**
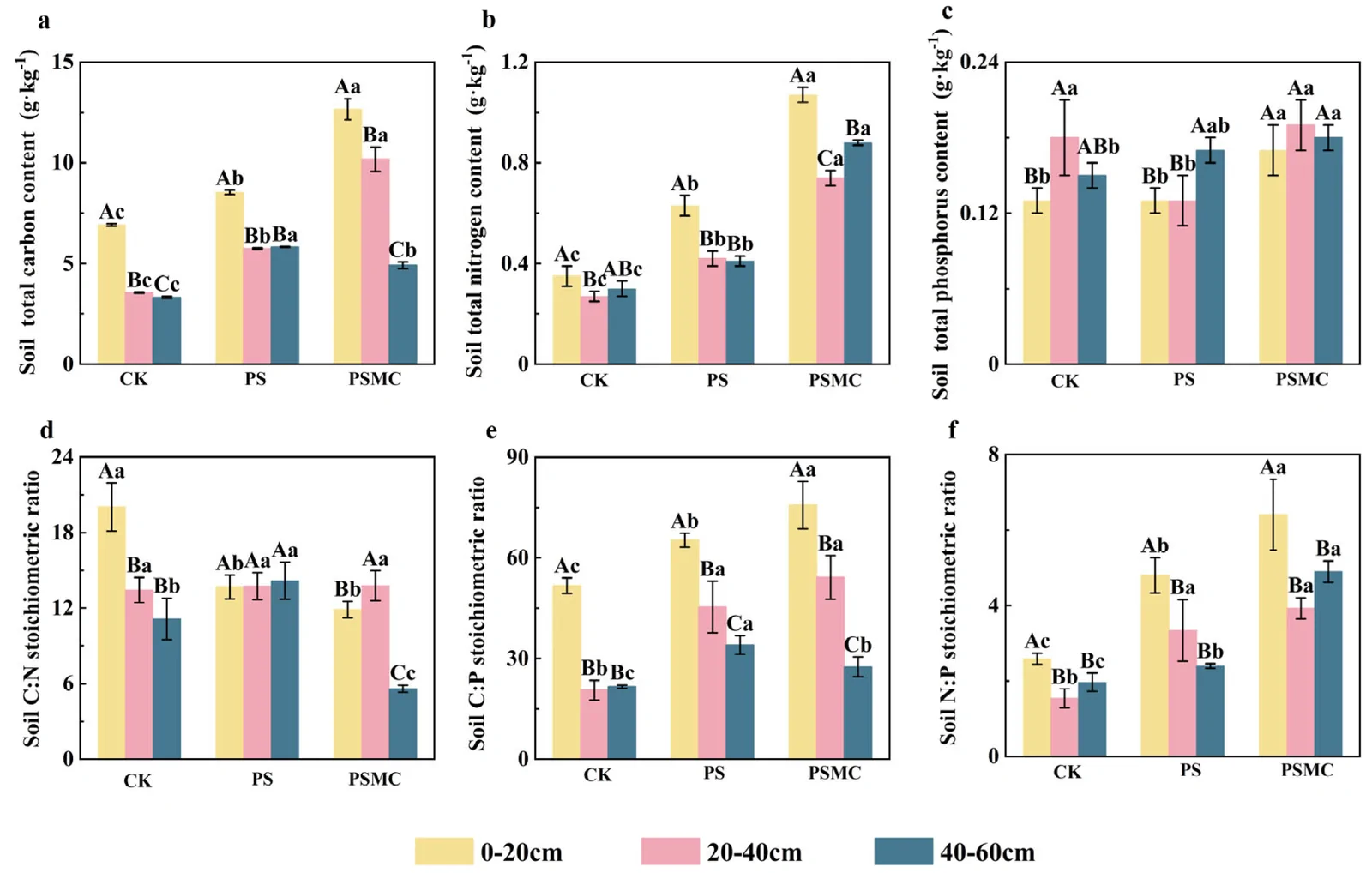
Soil nutrient content and stoichiometric ratio in different treatments. (**a**) Soil total carbon content. (**b**) Soil total nitrogen content. (**c**) Soil total phosphorus content. (**d**) Soil C:N stoichiometric ratio. (**e**) Soil C:P stoichiometric ratio. (**f**) Soil N:P stoichiometric ratio. Data are presented as mean ± SE. Different capital letters indicate significant differences between different soil depths within the same forest stands, while different lowercase letters indicate significant differences between different forest stands at the same soil depth (*p* < 0.05).

As shown in Figure 4a–f, soil C, N, and P concentrations as well as soil C:P and N:P ratios differed significantly among treatments in the 0–20 cm soil layer (*p* < 0.05). Soil C, N, and P concentrations in the PSMC stand were significantly greater than those in PS and CK stands, with C and N concentrations increased by 48.24% and 69.84%, 82.95% and 205.71%, relative to the two latter stands. Soil C:P and N:P ratios in the PSMC stand reached their maximum values, which were 46.45% and 147.49% higher than those in CK, respectively. In contrast, the soil C:N ratio was significantly lower in the PSMC stand than in CK, with the lowest value of 11.89 ± 0.65 recorded in the PSMC stand. A similar pattern was observed in 20–40 cm and 40–60 cm soil layers: soil C, N, and P concentrations as well as C:P and N:P ratios remained at relatively higher levels in the PSMC stand, while the C:N ratio was lower than others. Across all stand types, soil P concentration increased with increasing soil depth, whereas soil C and N concentrations and all stoichiometric ratios declined significantly. These results suggested that the PSMC stand maintained higher soil C, N, and P concentrations and higher C:P and N:P ratios across all soil layers, and that soil nutrient indices of the three stands exhibited significant vertical differentiation characteristics.

### 2.4. Correlation Analysis of Pinus massoniana Growth, Stand Biomass Accumulation, and Soil Stoichiometric Characteristics

**Figure 5:**
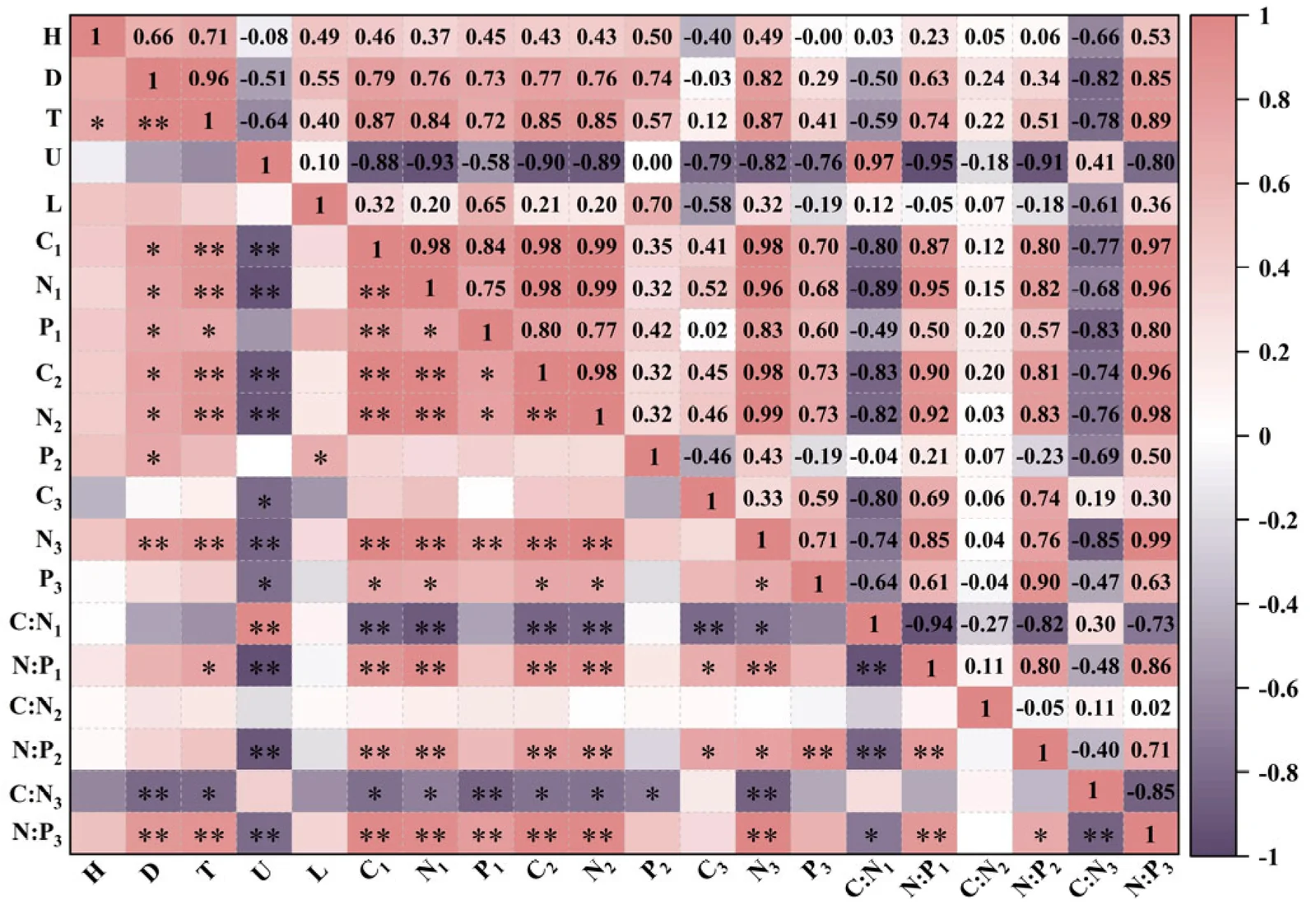
Correlations among *P. massoniana* growth, stand biomass accumulation and soil stoichiometric characteristics. Colors indicate the correlation coefficient (red, positive; purple, negative), and color intensity reflects the magnitude of the correlation. H: tree height of *P. massoniana*; D: diameter at breast height of *P. massoniana*; T: the biomass of the tree layer in the stand; U: the biomass of the understory vegetation layer in the stand; L: the biomass of the litter layer; C_1–3_, N_1–3_ and P_1–3_: soil carbon, nitrogen and phosphorus contents in each soil layer of 0–60 cm, respectively; C:N_1–3_ and N:P_1–3_: soil C:N and N:P ratios in each soil layer of 0–60 cm, respectively. “ * ” (*p* < 0.05), significant difference; “ ** ” (*p* < 0.01), extremely significant difference.

As shown in Figure 5, correlation analysis revealed that the DBH of *P. massoniana* positively correlation with soil C, N, and P concentrations in the 0–20 cm and 20–40 cm soil layers (*p* < 0.05). A highly significant positive correlation was also detected between DBH and soil N concentration as well as the soil N:P ratio in the 40–60 cm soil layer (*p* < 0.01), whereas DBH showed a significant negative correlation with the soil C:N ratio in the 40–60 cm layer. Moreover, tree-layer biomass showed a significant positive correlation with soil P concentration and the soil N:P ratio in the 0–20 cm layer. It also exhibited highly significant positive correlations with soil C and N concentrations in the 0–20 cm and 20–40 cm layers, as well as with soil N concentration and the N:P ratio in the 40–60 cm layer, whereas it was significantly negatively correlated with the soil C:N ratio in the 40–60 cm layer. In contrast, understory vegetation biomass was significantly negatively correlated with soil C and P concentrations in the 40–60 cm soil layer. It showed highly significant negative correlations with soil C and N concentrations and the soil N:P ratio in the 0–20 cm and 20–40 cm layers, as well as with soil N concentration and the N:P ratio in the 40–60 cm layer, whereas it presented a highly significant positive correlation with the soil C:N ratio in the 0–20 cm layer. Furthermore, litter-layer biomass exhibited a significant positive correlation with soil P concentration in the 20–40 cm soil layer.

## 3. Discussion

### 3.1. Effects of Understory Interplanting Patterns on Soil Nutrients and Stoichiometric Characteristics of Forest Stands

In this study, the soil N and P concentrations across the three stand types (CK, PS, and PSMC) were lower than the corresponding national averages in China (in 1.1 g·kg^−1^ and 0.7 g·kg^−1^, respectively), suggesting relatively low plant-available soil N and P for tree uptake in the study area [18]. Compared with the pure *P. massoniana* stand (CK), both understory interplanting patterns (PS and PSMC) significantly promoted soil C, N, and P concentrations across soil profile, suggesting that the introduction of broad-leaved species ameliorated soil nutrient conditions in *P. massoniana* plantations. This ameliorative effect is likely to be attributable to the increase of litter input, with accelerated decomposition rates and enhanced soil enzyme activities, thus fastening the nutrient cycling following the interplanting of broad-leaved trees [19,20]. Higher soil C, N, and P concentrations in the PSMC stand relative to the PS stand suggest that increased tree species richness may amplify the nutrient-enhancing effect. On the one hand, the multi-species interplanting pattern may have strengthened ecological complementarity between *P. massoniana* and broad-leaved species, creating a more favorable microenvironment for litter decomposition and nutrition release, which could promote greater nutrient accumulation and provide more organic matter and nitrogen sources [21]. On the other hand, differences in root spatial distribution and root exudate composition among diverse broad-leaved species can effectively activate the metabolic functions of phosphate-solubilising microbial communities, which may enhance the mobilisation of insoluble soil P and potentially alleviate phosphorus deficit in forest stands of Southern China [22,23].

Soil C, N, and P stoichiometric characteristics serve as critical indicators for elucidating soil nutrient cycling and nutrient limitation status [23,24]. The soil C:N ratio reflects the stability of soil organic matter and nitrogen transformation efficiency; the C:P ratio indicates the trade-off between carbon assimilation and P consumption and has been used to infer soil P-related processes; the N:P ratio is often discussed as a potential index associated with N and P supply for vegetation growth [25]. In this study, soil C:N ratios in the 0–20 cm layer of the PS and PSMC stands were lower than the national average for forest soils (13.7) [26]. The ratio was lower in the PSMC stand than that in the PS stand, whereas the CK stand exhibited a relatively higher value. These findings suggest potential differences in surface soil organic matter mineralisation rates among stands; the two interplanted stands, particularly the PSMC stand, may have a greater potential for nitrogen turnover compared with CK [27,28]. This pattern may arise from stronger microbial decomposition of carbohydrates and other labile compounds in litter. Nutrients released during decomposition enter the soil, promoting soil organic carbon accumulation and accelerating nitrogen transformation processes [29]. Furthermore, soil C:P and N:P ratios in most soil layers across the three stand types were lower than the national averages (61 and 5.2, respectively) [18]. An N:P ratio below 14 has previously been proposed as an indicator suggesting potential N limitation in similar ecosystems [23]. Soil N:P ratios in most layers of the PS and PSMC stands were significantly higher than those in CK (*p* < 0.05), suggesting that, if the proposed stoichiometric criterion applies here, interplanting with broad-leaved species may modify the relative balance of soil N and P. Interplanting broad-leaved species likely exerts a positive effect on soil N status, and this effect becomes more pronounced with increasing species diversity in interplanting systems [30]. In addition, soil C:P ratios across all three stand types were below 200, which has been proposed as an indicator of potential net mineralisation of soil P [31]. Notably, soil C:P ratios in the 0–20 cm soil layer under the PS and PSMC stands were significantly higher than those under the CK stand, and the PSMC stand exhibited a markedly higher soil C:P ratio than the P-S stand, because of the relatively higher carbon abundance relative to phosphorus in soil organic matter in broadleaved mixed stands. Generally, the multi species interplanting mode is hypothesised to exert a more significant promoting effect on soil phosphorus mobilisation than the single species interplanting mode.

### 3.2. Effects of Soil Nutrients and Stoichiometric Characteristics on Stand Biomass Allocation

In this study, the DBH of *P. massoniana* was significantly positively correlated with soil C, N and P concentrations in the 0–40 cm soil layer, which was also significantly positively correlated with soil N concentration and the N:P ratio in the 40–60 cm soil layer (*p* < 0.01). Furthermore, stand tree-layer biomass was highly significantly positively correlated with the N:P ratio in the 40–60 cm soil layer. Thus, the soil N:P ratio in the 40–60 cm soil layer may be one factor associated with stand tree-layer performance, and the increase in the N:P ratio in deeper soil layers (40–60 cm) may correlate with the accumulation of tree height, DBH and stand tree-layer biomass of *P. massoniana*. On the one hand, *P. massoniana* is a typical deep-rooted species, whose nutrient uptake and physiological metabolic processes are closely linked to nutrient status in the 40–60 cm soil layer [32]. On the other hand, deeper soil layers experience less external disturbance, such that the soil N:P ratio may more stably reflect the relative balance of soil nutrients shaped by long term pedogenesis and root activity. This was the main reason why interplanting broad-leaved species beneath *P. massoniana* stands elevated the soil N:P ratio in deeper soil layers, which may alter the nitrogen phosphorus stoichiometric ratio and is hypothesised to modify nutrient imbalance associated with relative nitrogen differences in the study area, improve tree nutrient uptake and utilisation efficiency, and ultimately promote height growth, radial growth, and stand tree-layer biomass accumulation of *P. massoniana* [33].

Furthermore, the PSMC stands significantly increased tree-layer leaf biomass in this study, because under-canopy interplanting modified the stand canopy structure, rendering in-stand light conditions a growth-limiting factor. During tree growth and development, plants allocate biomass in response to functional requirements and tend to allocate greater biomass fractions to organs that best mitigate growth-limiting constraints to sustain growth. To cope with light competition, trees preferentially allocate biomass to leaves. Greater leaf biomass improves photosynthetic areas, enabling trees to adapt to low-light environments [34]. Existing studies have demonstrated that under mixed-species management, plants adjust root biomass in response to the variations in soil moisture and nutrient regimes, thus developing a resource-acquisition strategy that differs from those of monoculture stands [35]. In this study, tree-layer root biomass was significantly higher in the PSMC stand compared to the CK and PS stands. It can be inferred that *P. massoniana* stands interplanted with *S. superba*, *C. hystrix*, and *M. macclurei* increased tree-species richness and allowed competitively dominant tree individuals to improve below-ground resource-acquisition efficiency via greater root biomass. This strategy expanded resource-acquisition space and supports optimal water and nutrient uptake [36]. Compared to CK, the PSMC stand promoted *P. massoniana* growth and biomass accumulation, whereas the PS stand exerted a relative inhibitory effect. Elevated litter diversity under multi-species mixed modes may partly explain the positive correlations between *P. massoniana* growth-biomass accumulation and soil nutrients within the PSMC stand. Existing studies have demonstrated that multi-species mixed stands substantially enhanced litter quantity and quality. Litter from different tree species varied in chemical composition and decomposition rate; mixed-species litter had more complex litter assemblages that facilitated soil organic-matter accumulation [37]. In addition, higher tree-species diversity may synergistically boost nutrient-cycling efficiency via niche complementarity and functional differentiation of microbial communities [37,38]. In contrast, *S. superba* grows rapidly within the PS stand and may generate intense light competition with *P. massoniana*, suppressing its growth. Meanwhile, allelopathic effects from *S. superba* can also inhibit root growth of *P. massoniana* [39]. However, the introduction of *C. hystrix* and *M. macclurei* under the PSMC mode may attenuate competitive inhibition imposed by a single broad-leaved species. Increased root biomass further improved nutrient availability in deeper soil layers, which was the main reason why tree-layer root biomass is hypothesised to alter nutrient conditions in deeper soil layers, which may partly explain why tree layer root biomass in the PSMC stand was significantly higher than that of the other two stand types.

Although understory vegetation biomass constituted a relatively small fraction of total biomass, it plays a vital role in nutrient cycling and biodiversity conservation [40]. In this study, interplanting broad-leaved species beneath *P. massoniana* stands significantly reduced understory vegetation biomass (*p* < 0.05), because of under-canopy interplanting modified the stand canopy structure, reduced light transmittance understory, and intensified light competition among plants, consequently decreasing the understory vegetation biomass [41]. Correlation analysis revealed that understory vegetation biomass exhibited a highly significant negative correlation with soil N:P ratios across soil layers. This correlation suggests that the introduction of broad-leaved species elevated soil N:P ratios in the 40–60 cm soil layer. Shifts in N:P ratios were observed here, but we cannot confirm that interplanting alleviated soil nitrogen deficiency or aggravated soil phosphorus deficit. As a deep-rooted tree species, *P. massoniana* absorbed water and nutrients from deeper soil layers via root plasticity [32]. Such vertical differentiation in root penetration depth between *P. massoniana* and interplanted broad-leaved species is hypothesised to contribute to changes in deep-soil nutrient stoichiometry. This result is consistent with our finding of higher tree-layer root biomass in the PSMC stand. However, we did not quantitatively measure the vertical distribution of fine roots for all tree species in this study. Further quantitative investigation of root penetration depths is required in future research to validate this mechanism. Furthermore, soil phosphorus concentration was mainly governed by differences in parent material and had little variations within the soil profile. Understory vegetation, with roots predominantly confined to shallow soil layers, was more sensitive to phosphorus supply status under the relatively phosphorus-poor red-soil conditions of the study area. Higher soil N:P ratios are recorded here, but this alone cannot confirm relative phosphorus limitation that suppresses understory vegetation growth [42]. Accordingly, the combined pressures of above-ground light competition and below-ground nutrient competition may have contributed to significant reduction of understory vegetation biomass in the PS and PSMC stands.

## 4. Materials and Methods

### 4.1. Description of the Experimental Site

**Figure 6:**
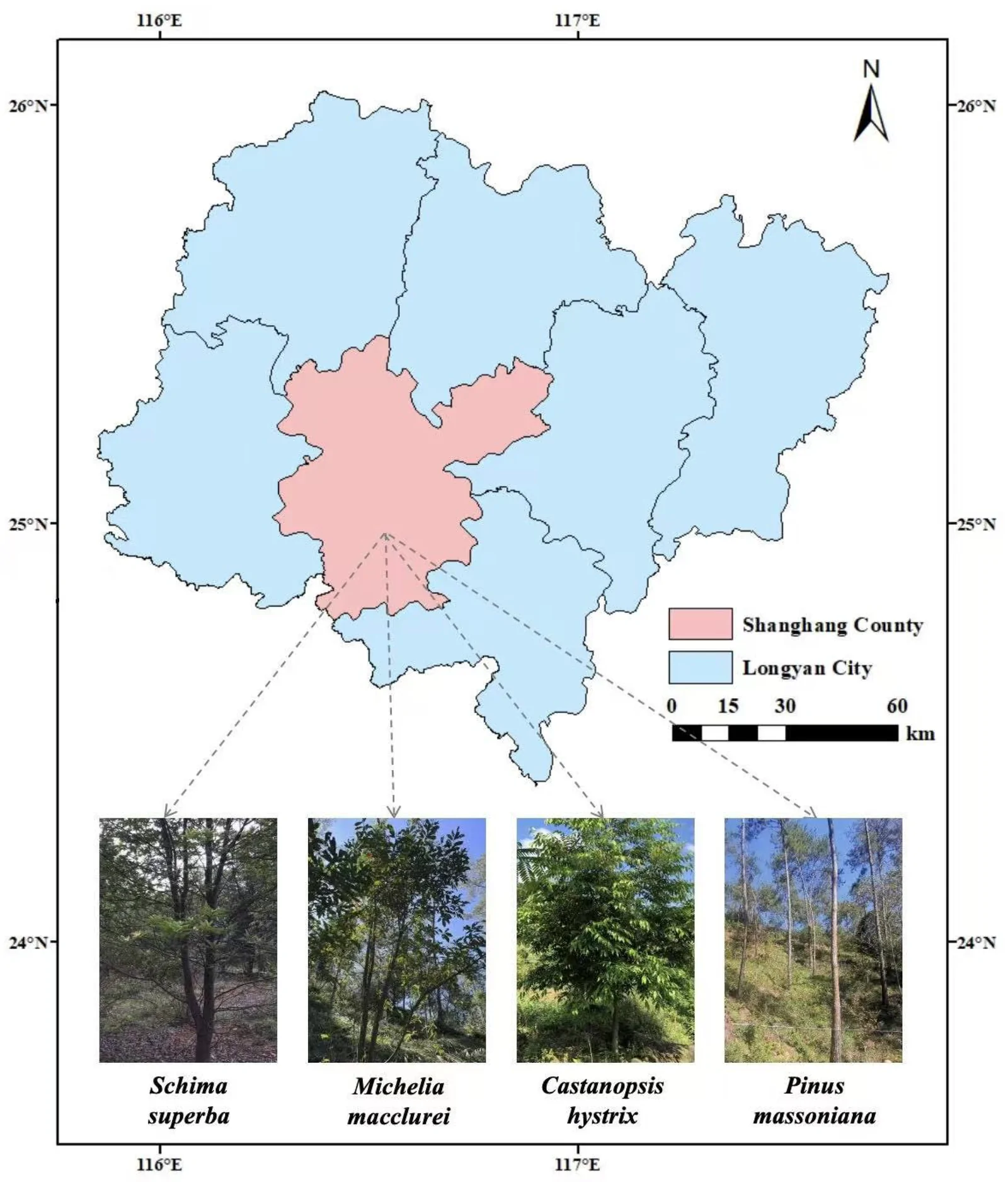
Schematic map of the study area location.

As shown in Figure 6, The experimental site is located at Baisha State-Owned Forest Farm, Shanghang County, Fujian Province, China (25°01′ N, 116°20′ E). This region has a subtropical oceanic monsoon climate, which was characterised by ample hydrothermal conditions. The mean annual precipitation range is 1600–1800 mm, mean annual temperature is approximately 20 °C, the frost-free period is 295 d, and annual sunshine duration totals 1921 h. The soil is typical hilly red soil, characterised by a relatively thin humus horizon and intense weathering-leaching processes. Vegetation within this forest area is dominated by plantations and natural forests. Major tree species include Chinese fir (*Cunninghamia lanceolata*), moso bamboo (*Phyllostachys edulis*), and *P. massoniana*. Understory vegetation is rich in species diversity, with representative species including *Mussaenda pubescens*, *Ilex pubescens*, and *Ligustrum lucidum*.

### 4.2. Experimental Design

A pure *P. massoniana* plantation established in 1992 (1-year-old seedlings at planting) was selected for this study. Uniform tending-thinning operations were carried out in 2019 in the 27-year-old stand, with residual stand density maintained at 150 trees·hm^−2^. In the same year, uniformly selected one-year-old seedlings of three native broad-leaved tree species (*S. superba*, *M. macclurei*, and *C. hystrix*) with homogeneous growth, sound development, free of pests and diseases, and consistent initial seedling quality were planted beneath the canopy to set up an under-canopy interplanting experiment at a planting spacing of 4 m × 5 m. Two interplanting modes were set up: *P. massoniana* interplanted with *S. superba* (denoted by PS), and *P. massoniana* interplanted with *S. superba* + *M. macclurei* + *C. hystrix* (denoted by PSMC). A pure *P. massoniana* stand without interplanting was used as the control (CK). Within the PSMC treatment, equal numbers of seedlings were planted the three broad-leaved species. Previous studies support our species selection. Chen et al. [43] compared the growth of pure *P. massoniana* stands, pure S. superba stands, and *P. massoniana*–*S. superba* mixed stands and found that mixed stands exhibited higher stand volume and total biomass than monocultures. Guo et al. [44] reported that mixtures of *P. massoniana* with *M. macclurei* and *C. hystrix* are suitable combinations for large-diameter timber cultivation. These three native broad-leaved species are well adapted to the understory environment of plantations. Therefore, we selected these for the underplanting experiment. In December 2021, three independent stands were selected for each of the three interplanting treatments. One 20 m × 20 m standard plot was established within each independent stand, resulting in a total of nine plots. The plots were randomly allocated across the experimental area, with a minimum distance of ≥20 m between adjacent plots to avoid spatial autocorrelation. All plots had homogeneous tree distribution and comparable site conditions (Table 2). No artificial clearing or tending operations were performed on the understory shrub and herb layers across all experimental stands. In October 2022, the diameter at breast height (DBH) and tree height within the 9 plots was measured. The counts and densities of *P. massoniana* and broad-leaved trees for each plot are presented in Table 3, and the full individual tree measurement data for all plots are provided in the Supplementary Materials. Canopy photographs were captured using a canopy analyzer (Yaxin-1201, Yaxin Liyi, Beijing, China) for calculating canopy closure.

### 4.3. Experimental Methods

#### 4.3.1. Total Tree-Layer Biomass

Total tree-layer biomass represents the summed biomass of individual organs (leaves, branches, trunks, roots), including *P. massoniana* and interplanted broad-leaved tree species. The biomass of *P. massoniana* was calculated based on the DBH and tree height of each tree in the sample plots. Individual-tree allometric equations were used to estimate the biomass of each tree (kg tree^−1^). The biomass values of all *P. massoniana* trees within each plot were summed to obtain the total plot biomass, which was then converted to biomass per hectare (t ha^−1^) according to the plot area. Organ biomass for *P. massoniana* was estimated via species-specific allometric biomass models [45], as shown below:

(1) $${\mathrm{Leaves}\;\mathrm{biomass}\;W}_{1}=W_{5}\times \frac{f_{1}}{1+f_{1}+f_{2}+f_{3}}$$

(2) $${\mathrm{Branches}\;\mathrm{biomass}\;W}_{2}=W_{5}\times \frac{f_{2}}{1+f_{1}+f_{2}+f_{3}}$$

(3) $${\mathrm{Trunks}\;\mathrm{biomass}\;W}_{3}=\frac{W_{5}}{1+f_{1}+f_{2}+f_{3}}$$

(4) $${\mathrm{Roots}\;\mathrm{biomass}\;W}_{4}=0.008112D^{2.6905}$$

(5) $${\mathrm{Aboveground}\;\mathrm{biomass}\;W}_{5}=0.099488D^{2.40859}$$

*f*_1_, *f*_2_, and *f*_3_ are, respectively:

(6) $$f_{1}=0.9871D^{-0.81602}$$

(7) $$f_{2}=0.51699D^{-0.2728}$$

(8) $$f_{3}=0.31246D^{-0.3974}$$

where W_1_–W_5_ indicate the biomass of individual tree organs (kg·tree^−1^), and D denotes tree diameter at breast height (DBH, cm); $f_{1}$, $f_{2}$, and $f_{3}$ are the ratio functions of leaves, branches, and trunks, respectively, which relative to wood biomass is 1.

The biomass of interplanted broad-leaved species (*S. superba*, *M. macclurei*, and *C. hystrix*) was measured using the whole-tree excavation method. In this experiment, one standard tree representing the average size of each broad-leaved species was selected and felled within each sample plot. Under the two interplanting forest treatments, a total of three standard trees were harvested for each broad-leaved species. To account for variations in tree size among individuals, the selected standard trees had DBH and tree height values closest to the mean DBH and mean height of the target species in the corresponding plots (Table 4). The harvested tree was divided into stem, branch, leaf, and root tissues, and the fresh weight of each tissue was recorded. All samples were oven-dried to a constant weight for subsequent biomass calculation. The total biomass of an individual standard tree was the sum of biomass across all its organs. The stand biomass of each broad-leaved species in each plot was calculated by multiplying the individual standard tree biomass by the corresponding tree density of the species. However, this estimation method ignores the size differentiation among individual trees, which may have introduced certain biases to stand biomass estimates.

#### 4.3.2. Biomass of Understory Vegetation and Forest Litter

In each standard plot, three 2 m × 2 m quadrats were randomly set up in a triangular pattern for shrub sample collection. One 1 m × 1 m sub-quadrat was nested within each 2 m × 2 m quadrat, yielding a total of three sub-quadrats for herbaceous vegetation and forest litter sampling. The fresh weights of above-ground and below-ground components of shrubs, herbs, and forest litter were measured. All samples from within each quadrat were collected, placed in labeled paper bags, and transported to the laboratory. In the laboratory, litter samples were cleaned to remove stones, soil, and roots, oven-dried at 65 °C to constant weight, and weighed to estimate litter biomass per unit area. All samples were oven-dried to a constant weight, and the biomass of understory vegetation and forest litter layers was calculated accordingly.

#### 4.3.3. Soil Sampling and Preprocessing

Within each standard quadrat of woodlands under three interplanting patterns, three soil sampling points were arranged along the diagonal of the quadrat to collect soil samples in layers between *P. massoniana* and the understory interplanted tree species. At each point, soil was collected from depths of 0–20, 20–40, and 40–60 cm using a soil auger or by excavating a small soil profile. For each soil layer, equal amounts of soil from the three points were thoroughly mixed to form one composite sample per plot and per layer. All soil samples were air-dried, cleaned to remove impurities, and sieved for subsequent laboratory analysis. Soil total carbon (TC) and total nitrogen (TN) were measured using an elemental analyzer (Vario Max, Elementar, Germany). Soil total phosphorus (TP) was quantified via hydrofluoric–perchloric acid digestion and the molybdenum–antimony-scandium colorimetric method. The C:N, C:P, and N:P ratios were first calculated as mass ratios for each individual soil sample and then averaged.

### 4.4. Statistical Analysis

All data were statistically processed using Microsoft Excel 2022, SPSS 26.0, and R 4.6.0. A two-way analysis of variance (ANOVA) was applied to examine the interactive effects of stand type and soil layer on soil carbon, nitrogen, phosphorus and their corresponding stoichiometric characteristics. One-way ANOVA coupled with Duncan’s multiple range test (*p* < 0.05) was performed to detect differences in *P. massoniana* growth and stand biomass allocation. Pearson correlation analysis was conducted to quantify the correlations between soil nutrient factors and stand growth indices. All graphs and statistical figures were plotted using Origin 2026 software.

## 5. Conclusions

In this study, two understory interplanting treatments of *P. massoniana* stand, namely, *S. superba* monoculture interplanting (PS) and mixed interplanting of *S. superba*, *C. hystrix*, and *M. macclurei* (PSMC), significantly promoted soil carbon, nitrogen, and phosphorus concentrations, as well as soil C:P and N:P stoichiometric ratios across all soil layers. Correlation analysis showed that the soil N:P ratio in the 40–60 cm deep soil layer exhibited a significant positive correlation with the tree height, DBH, and tree-layer biomass of *P.massoniana*, whereas it was significantly negatively correlated with understory vegetation biomass. Furthermore, the PSMC significantly improved the tree height and DBH of *P. massoniana*, facilitated tree-layer biomass accumulation, and substantially decreased understory vegetation biomass. In contrast, the PS interplanting mode reduced tree height, DBH, and biomass of the tree layer, understory vegetation layer, and litter layer compared to the pure *P. massoniana* stand (CK). Overall, the PSMC interplanting mode had an optimal performance in enhancing stand productivity and altering measured soil nutrient concentrations and stoichiometry. We hypothesise that such changes in soil elemental concentrations and stoichiometry may be linked to soil nutrient conditions, although total N and P concentrations alone cannot confirm shifts in plant available nutrient supply or nutrient limitation status. This finding offers a scientific basis for the sustainable management and quality improvement of *P. massoniana* plantations in southern China, especially for pre-mature pure *Pinus massoniana* stands growing on red soil within a subtropical oceanic monsoon climate.

## Figures and Tables

**Table 1:** Biomass and distribution of tree layer in different stands.

Forest Type	Leaf Biomass (t·hm^−2^)	Branch Biomass (t·hm^−2^)	Trunk Biomass (t·hm^−2^)	Root Biomass (t·hm^−2^)	Above Ground Biomass (t·hm^−2^)	Total Biomass (t·hm^−2^)
CK	2.44 ± 0.23 b	8.23 ± 1.07 b	43.98 ± 6.41b	11.78 ± 1.89 b	54.65 ± 7.71 b	66.43 ± 9.60 b
PS	3.44 ± 0.89 ab	8.17 ± 0.67 b	41.88 ± 4.20 b	11.62 ± 1.10 b	53.49 ± 5.23 b	65.11 ± 6.31 b
PSMC	3.83 ± 0.89 a	11.87 ± 0.68 a	63.41 ± 4.23 a	17.76 ± 1.21 a	79.11 ± 4.97 a	96.87 ± 6.18 a

Data are presented as mean ± SE. Different lowercase letters indicate significant differences between different forest stands (*p* < 0.05).

**Table 2:** Basic information of sample plots.

Forest Type	Density (Plants·hm^−2^)		Altitude (m)	Slope (°)	Aspect	Canopy Density
	*P. massoniana*	Broad-Leaved Trees				
CK	150	/	342–374	28–32	Southwest	0.6
PS	150	500–600	352–397	24–29	Southwest	0.7
PSMC	150	500–600	344–366	25–28	Southwest	0.6

/ = No understory broad-leaved tree interplanting.

**Table 3:** Number and density of *P. massoniana* and broad-leaved trees in each plot.

Plot (20 m × 20 m)	*P. massoniana*		Broad-Leaved Trees	
	Number (Trees)	Density (Trees·hm^−2^)	Number (Trees)	Density (Trees·hm^−2^)
CK-1	7	175	0	0
CK-2	7	175	0	0
CK-3	6	150	0	0
PS-1	7	175	20	500
PS-2	7	175	21	525
PS-3	6	150	20	500
PSMC-1	7	175	24	600
PSMC-2	7	175	23	575
PSMC-3	6	150	25	625

**Table 4:** Height and DBH of broad-leaved species.

Forest Type	Broad-Leaved Species	Mean Height (m)	Mean DBH (cm)
PS	*S. superba*	3.70 ± 0.11	3.17 ± 0.16
PSMC	*S. superba*	3.26 ± 0.15	2.71 ± 0.19
	*M. macclurei*	4.24 ± 0.12	3.60 ± 0.17
	*C. hystrix*	4.49 ± 0.11	3.62 ± 0.65

Data are presented as mean ± SE.

## Data Availability

All data supporting this study are provided in the article. Any further questions may be directed to the corresponding author.

## Supplementary Materials

The following supporting information can be downloaded at: https://www.mdpi.com/article/10.3390/plants15213456/s1.

## Abbreviations

The following abbreviations are used in this manuscript:

PS	*P. massoniana* interplanted with *S. superba*
PSMC	*P. massoniana* interplanted with *S. superba* + *M. macclurei* + *C. hystrix* (PSMC)
CK	Pure *P. massoniana* stand without interplanting
H	Tree height of *P. massoniana*
D	Diameter at breast height of *P. massoniana*
T	The biomass of the tree layer in the stand
U	The biomass of the understory vegetation layer in the stand
L	The biomass of the litter layer
C_1_	Soil carbon contents in 0–20 cm
C_2_	Soil carbon contents in 20–40 cm
C_3_	Soil carbon contents in 40–60 cm
N_1_	Soil nitrogen contents in 0–20 cm
N_2_	Soil nitrogen contents in 20–40 cm
N_3_	Soil nitrogen contents in 40–60 cm
P_1_	Soil phosphorus contents in 0–20 cm
P_2_	Soil phosphorus contents in 20–40 cm
P_3_	Soil phosphorus contents in 40–60 cm
C:N_1_	Soil C:N stoichiometric ratio in 0–20 cm
C:N_2_	Soil C:N stoichiometric ratio in 20–40 cm
C:N_3_	Soil C:N stoichiometric ratio in 40–60 cm
N:P_1_	Soil N:P stoichiometric ratio in 0–20 cm
N:P_2_	Soil N:P stoichiometric ratio in 20–40 cm
N:P_3_	Soil N:P stoichiometric ratio in 40–60 cm
